# Estimating Temporal Causal Interaction between Spike Trains with Permutation and Transfer Entropy

**DOI:** 10.1371/journal.pone.0070894

**Published:** 2013-08-05

**Authors:** Zhaohui Li, Xiaoli Li

**Affiliations:** 1 Institute of Information Science and Engineering, Yanshan University, Qinhuangdao, China; 2 National Key Laboratory of Cognitive Neuroscience and Learning, Beijing Normal University, Beijing, China; McGill University, Canada

## Abstract

Estimating the causal interaction between neurons is very important for better understanding the functional connectivity in neuronal networks. We propose a method called normalized permutation transfer entropy (NPTE) to evaluate the temporal causal interaction between spike trains, which quantifies the fraction of ordinal information in a neuron that has presented in another one. The performance of this method is evaluated with the spike trains generated by an Izhikevich’s neuronal model. Results show that the NPTE method can effectively estimate the causal interaction between two neurons without influence of data length. Considering both the precision of time delay estimated and the robustness of information flow estimated against neuronal firing rate, the NPTE method is superior to other information theoretic method including normalized transfer entropy, symbolic transfer entropy and permutation conditional mutual information. To test the performance of NPTE on analyzing simulated biophysically realistic synapses, an Izhikevich’s cortical network that based on the neuronal model is employed. It is found that the NPTE method is able to characterize mutual interactions and identify spurious causality in a network of three neurons exactly. We conclude that the proposed method can obtain more reliable comparison of interactions between different pairs of neurons and is a promising tool to uncover more details on the neural coding.

## Introduction

The question of how neurons interact has gained considerable attention over the years [1], [2]. Traditional methods emphasize the strength of pairwise connections, i.e. the degree of similarity or dissimilarity between two spike trains, such as the cost-based metric [3], the van Rossum distance [4], the event synchronization method [5], the cross correlation [6] and the interspike interval distance [7]. However, they cannot offer any insights into the information flow from one neuron to another because of their symmetric properties. In fact, fundamental knowledge about the causal interaction between spike trains is crucial for deeper insights into the neural coding [8], [9]. In recent years, analyzing the causality between recordings from different neurons is of increasing interest [10]–[15].

Information theoretic measures are inherently non-linear and do not require a model of the interaction, thus they are widely applied for the causality detection between neural signals [16]–[20]. For continuous-valued signals, e.g. electroencephalography (EEG) and local filed potential (LFP), recent works showed that transfer entropy (TE) is a good method for estimating the causal interaction [19], [21], which can quantify the fraction of information in the history of a time series flowed to the future of another time series when the latter is already known. The TE has also applied to analyzing spike trains, and referred as normalized transfer entropy (NTE), so as to evaluate the information transferred between auditory cortical neurons [15], the NTE can detect the asymmetry in the interaction between neurons, namely the causality. The symbolic transfer entropy (STE) is an improvement of the TE, which makes use of the technique of symbolization (i.e. permutation) to quantify the information flow between time series [9], [20]. Another new information theoretic method is called as permutation conditional mutual information (PCMI), which can estimate the coupling direction between neural series based on the ordinal structure of data [16]. The PCMI has also been employed to characterize the causal interaction between spike trains, which is more immune to noisy spikes than the TE [13]. By means of the discretizing procedure in the PCMI calculation, the STE also can be employed to analyze spike trains. A brief description of these three methods is given in File S1.

Unfortunately, we find that the amount of information flow estimated with the PCMI and STE is greatly influenced by the firing rate of neurons. Specifically, increasing firing rate brings about spurious increase of the information flow. The NTE is superior to them but it is not robust for estimating the time delay of causality between spike trains. This will be illustrated in the following sections. In this paper, we integrate permutation and transfer entropy to propose normalized permutation transfer entropy (NPTE) for characterizing the causal connectivity between spike trains, with the intention to reduce the influence of firing rate and improve the robustness of time delay estimation between the spike trains. The performance of the approach is evaluated using an Izhikevich’s neuronal model by comparing with the NTE, PCMI and STE methods. To further demonstrate the ability of NPTE to describe mutual interactions and identify spurious causalities, the Izhikevich’s cortical network model is employed to construct a network with simulated biophysically realistic synapses.

## Materials and Methods

### Normalized Permutation Transfer Entropy

**Figure 1:**
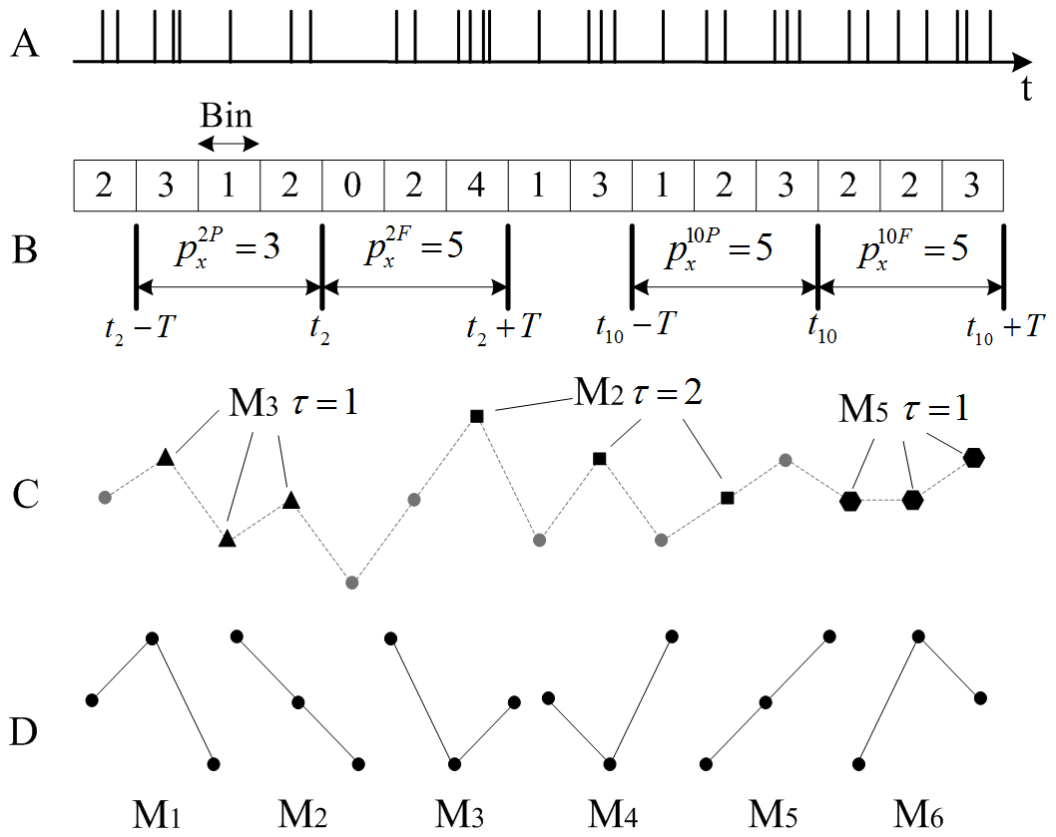
Extracting motifs from a simulated spike train. (A) A simulated spike train 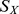. (B) Discretizing the spike train by counting spikes in each bin and the generation of 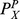 and 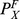. (C) Some motifs contained in the discretized sequence. (D) All motifs for the order 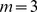
(3! = 6 different motifs), which are identical to those defined in Ref. [23].

Firstly, the spike trains are discretized to sequences of non-negative integers, which denote the number of spikes that occurred in each bin 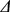 [22]. Generally, there are some ordinal patterns of the integers in the discretized spike trains. The scheme is illustrated in Figures 1(A–C). The ordinal patterns are also referred as motifs that are defined in Ref. [23]. The number of total motifs is equal to *m*!, i.e. the number of data points in each motif. The motifs for 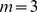 are shown in Figure 1(D). In case of equal values in motifs the arrangement is determined by the index of data involved, which ensures that they are uniquely mapped into one of the *m*! possible permutations. For example, as illustrated in Figure 1(C), the motif that includes three points denoted by solid hexagons is assigned a label of M5. Following Ref. [16], we choose the order 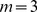 in this study. The rational for this choice is in the following: to ensure that every possible joint ordinal motif occurs at least once in the two neural signals of length *L*, the order *m* needs to satisfy the condition 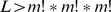. Thus, if a large value of *m* is selected, it will need a large amount of data and lose the temporal information in the data. On the other hand, only very few distinct states are available for 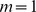 or 2. Thus, 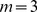 is appropriate for the calculation of NPTE. In addition, it should be pointed out that if the dynamics are over large time scales and there are sufficient data a lager *m* should be tried. Another important parameter for permutation analysis is the lag 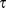, which denotes the number of data points spanned by each section of the motif [23]. The selection of this value will be discussed in Sec. 4.

Secondly, the transfer entropy based on the permutation analysis is defined in the following. Suppose two spike trains 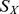 and 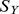 are given. Instead of the number of spikes, we use the ordinal patterns to calculate the information transferred from 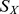 to 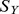. Let 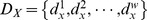 and 
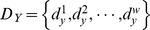
 denote the discretized spike trains of 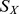 and 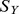, where 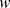 is the total number of bins within the recorded time interval. Let 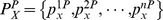 and 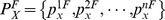 denote the motif numbers in 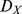, where 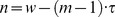, 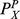 and 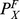 denote the ordinal patterns of 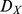
falling in the past time interval and the upcoming time interval respectively, 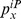 is the motif number of three data points: 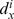, 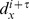, 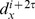, 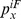 is the motif number of three data points: 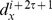, 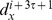, 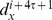, 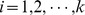, 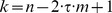. The generation of 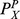 and 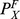 for a simulated spike train 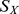 is illustrated in Figures 1(A–C). 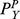 and 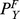
can be obtained in the same way. Then, the distribution of 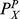, 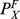, 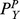 or 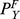 is discrete and the permutation transfer entropy (PTE) from 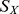 to 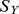 can be defined as
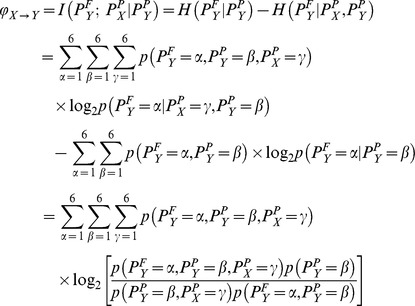
where 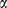, 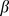, and 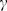
denote the motif number, which range from one to six for order 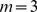.

Lastly, it is necessary to remove the bias that makes the PTE value tend to drift upwards as a function of the bin. When the bin is large, the joint distribution of 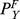, 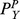, and 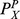 will be broad and sparse, which induces a spurious increase of the PTE from 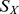 to 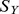 [15]. We use the procedure introduced in Ref. [15] to reduce this influence, including removal of the shuffled estimation and normalization. Randomly shuffling the interspike intervals in 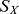 disconnects 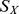 and 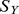 without altering the interspike intervals distribution of either. The shuffled estimation of the PTE is an average of results obtained on 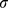
(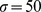 in this study) trials and is denoted as 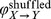. Furthermore, the remainder should be normalized by the conditional entropy in 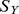
of its future on its past. Thus, the normalized permutation transfer entropy can be defined as
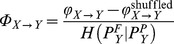
which represents the fraction of permutation information in 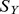
not predicted by its own past but predicted by the past of 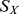. We take the maximum over all values obtained with different bins as the final NPTE estimation. Note that the value of NPTE may be negative at some time scales. To restrict the NPTE in the interval [0 1], we set up: 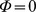 if 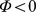. The directionality index 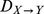
is defined in the following,



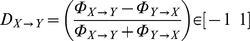


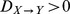
 means that the spike train 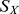 drives 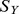, and 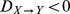, the spike train 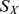 is driven by 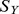. 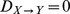 means that the interaction between 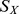 and 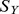 is symmetrical. Note that if 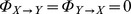, there is no temporal causal relationship between the two spike trains.

### Simulations

To evaluate the performance of the proposed method, Izhikevich’s neuronal model is employed to generate spike trains for simulation analysis, which integrates the computational efficiency of integrate-and-fire neurons with the biological plausibility of Hodgkin-Huxley-type dynamics [24]. The model is in the following,
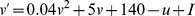


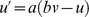
with the auxiliary after-spike resetting,



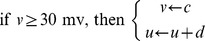
where *v* is the membrane potential of the neuron and *u* is the membrane recovery variable. The parameter *a* describes the time scale of *u*, *b* describes the sensitivity of *u* to sub-threshold fluctuations in *v*, *c* is the spike reset values of *v* and *d* is the spike reset value of *u*. They are set as: 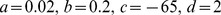. The injected current *I* is set to be a normally distributed random Gaussian variable, whose amplitude determines the firing rate *R* of the simulated spike trains. Utilizing this model, two independent spike trains 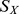 and 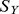 are firstly generated. Then we set up the causality between two spike trains by means of the method introduced in Ref. [15]. That is, a proportion 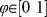 of spikes in 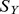 are replaced by the same number spikes that are picked up randomly from the 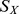 and delayed by some time (denoted by *D*). Through this replacement, the 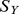 will contain the causal information from the 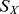, which is proportional to 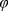. The spikes in 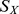 which are used to generate the causality and their delayed copies in the 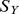 are referred as ‘causal spikes’ in the following sections. The duration of the simulated spike train is 10s through this study except for the performance test on data length.

To simulate biophysically realistic synapses, we use Izhikevich’s cortical network model [25] to construct a simulated network, which has been widely used in spike train studies [26]–[29]. According to Izhikevich, the model has many realistic features including firing patterns, synaptic delays and spike-timing dependent plasticity (STDP). By adjusting the parameters (i.e. *a*, *b*, *c* and *d* in the above neuronal model), many firing patterns observed in cortical neurons can be generated, including regular spiking (RS), intrinsically bursting (IB) and fast spiking (FS). The MATLAB code to implement this simulation is based on Ref. [25] and modified slightly as described below. The network model contains two types of neurons: 16 RS neurons (excitatory neurons) with parameters [*a*, *b*, *c*, *d*] = [0.02, 0.2, −65, 8] and 4 FS neurons (inhibitory neurons) with parameters [*a*, *b*, *c*, *d*] = [0.1, 0.2, −65, 2]. Each neuron has three delayed postsynaptic connections to other neurons. Excitatory neurons connect to any neurons with delays uniformly distributed in time interval ranging from 1 ms to 30 ms, while inhibitory neurons only connect to excitatory neurons with a fixed delay of 6 ms. In Eq. 4, *I* represents the total synaptic input arriving at a neuron, containing thalamic input and inputs from other cortical neurons. Every thalamic input has a synaptic weight of 20 mV that delivered at random times. The weights of inputs from excitatory neurons are initially set as 6 mV, however the weights of inputs from inhibitory cortical neurons are always set as 5 mV. Then, these excitatory weights are evolved by STDP in the manner in the following: synaptic inputs arrived before a postsynaptic spike with the derivatives of their weights increase, while synaptic inputs arrived after a postsynaptic spike decrease, where the increment or decrement decays exponentially with a time constant of 20 ms. The simulation runs for two hours, but only the last one minute of the output are used to evaluate the NPTE algorithm to avoid model transients. Because of synaptic plasticity, excitatory connections present variant synaptic weights ranging from 0 to 10 mV. We take the mean of weights within the last one minute as the final weight of each excitatory connection. In this manner, we get spike timings of 20 neurons, as well as the synaptic weights and time delays of the connections between them.

## Results and Discussion

### Parameter Choice

**Figure 2:**
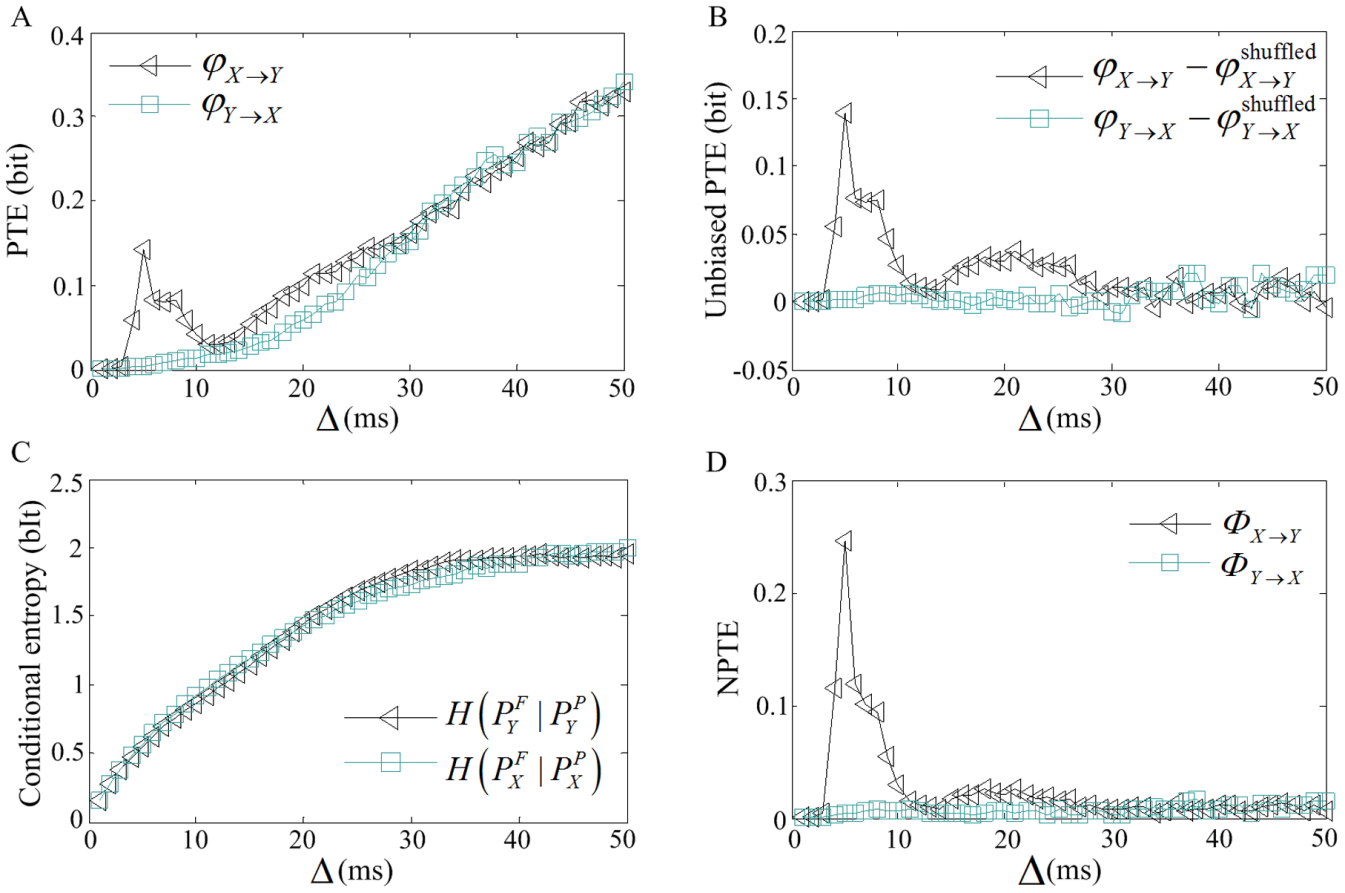
Removal of the bias and normalization of the PTE. (A) Permutation transfer entropy. (B) Unbiased permutation transfer entropy. (C) Conditional entropy. (D) Normalized permutation transfer entropy.

Before discussing the parameter choice, we first investigate the necessity of removing bias and the effect of normalization. Given 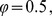 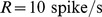 and 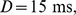 spike trains 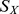 and 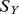 are generated. In the calculation, the lag is set as 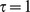. As shown in Figure 2(A), when 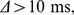 the PTE value increases with the increase of bin, which is caused by the sparse joint distribution of 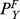, 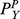, and 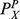. As can be seen in Figure 2(B), removal of this bias eliminates the spurious increase and the PTE stays close to 0 for large bins. Figure 2(C) shows that the amount of information available in 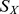 and 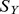
also increases with the increase of the bins. In Figure 2(D), the normalization of PTE by the conditional entropy highlights the main peak at 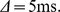


**Figure 3:**
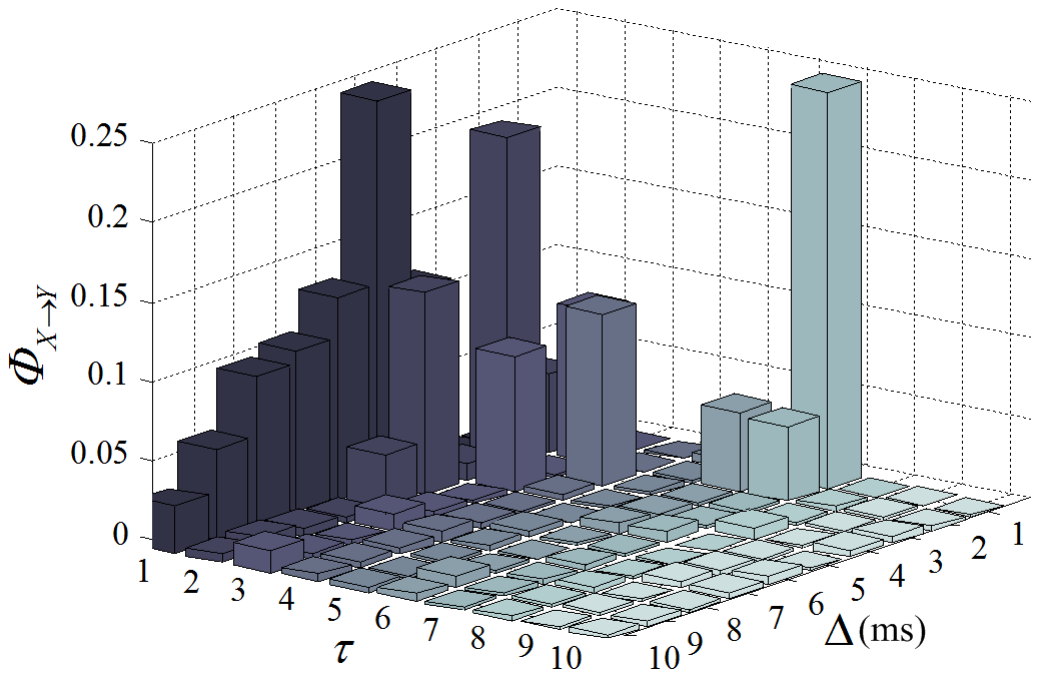
NPTE values for different 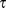 and 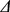.

Then, we study the parameter choice for the NPTE before its application. The bin 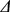 is used to discretize spike trains. In Figure 2(D), there is a peak for 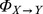 at 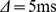 which is related to the delay of the model, meaning that the most information is transferred from the 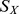 to the 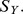 For a small 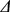
(e.g. 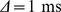), the 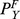 and the 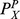 are independent because they cannot involve the causal spikes. For a big 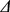 (e.g. 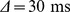), a pair of two causal spikes are located in one time interval and the causal relationship is destroyed. Actually, the product of order and the ‘peak bin’ (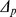, where the peak value is present) indicates the time delay between two causal spike trains. Thus it is necessary to chose many bins to investigate the information transferred at all delay times for real data. Another parameter, the lag 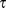
is the distance between adjacent two points in each motif. Figure 3 shows the NPTE 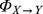 for different 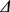 and 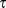. Clearly, there are obvious peaks for 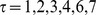, while the NPTE values for 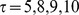 are always close to zero. The underlying reason is that the transferred information at different time scales is proportional to the extent that the two causal spikes are involved in the motifs which generate 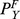 and 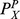. It is believed that there always exists a obvious peak for 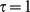 in the curve of NPTE values as a function of 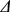. Thus, herein 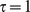 is suggested for the NPTE calculation.

### Comparison with other Methods

The methods mentioned in this paper are all able to estimate the coupling strength and time delay of the causal interaction between spike trains. Although they have similar characteristics, the proposed NPTE measure is superior to the others. The comparison is performed in the following four aspects.

#### Variation with coupling strength

**Figure 4:**
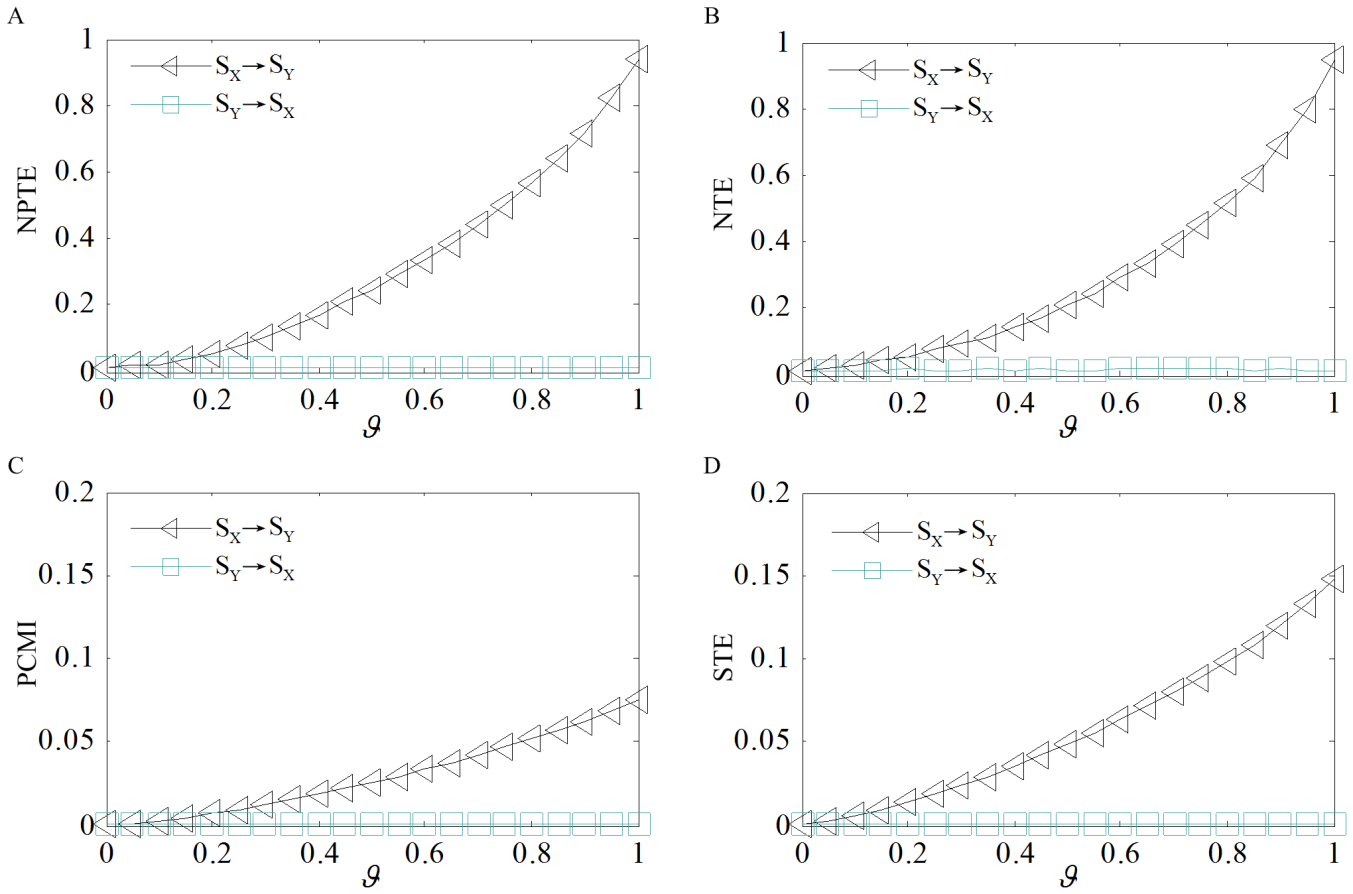
Variation of estimated values with coupling strength between spike trains. (A) NPTE. (B) NTE. (C) PCMI. (D) STE.

Herein, we demonstrate the variation of estimated values with the coupling strength between two spike trains which is determined by the proportion 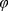. Given 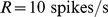
and 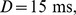 spike trains 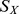 and 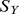
are generated. The 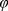 ranges from 0 to 1 with a step of 0.05. As shown in Figures 4(A–D), the estimation from 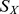 to 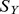
is nonlinearly related to 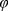, including NPTE, NTE, PCMI and STE. On the other hand, the estimation from 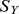 to 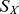 is always close to 0 because there is no causality in this direction. Specifically, NPTE and NTE are normalized metrics and they take values from 0 to 1 as the strength increases. Although PCMI and STE are not normalized, they can reflect the strength under certain firing rate. That is, the four methods are all able to describe the coupling strength between spike trains.

#### Influence of data length

**Figure 5:**
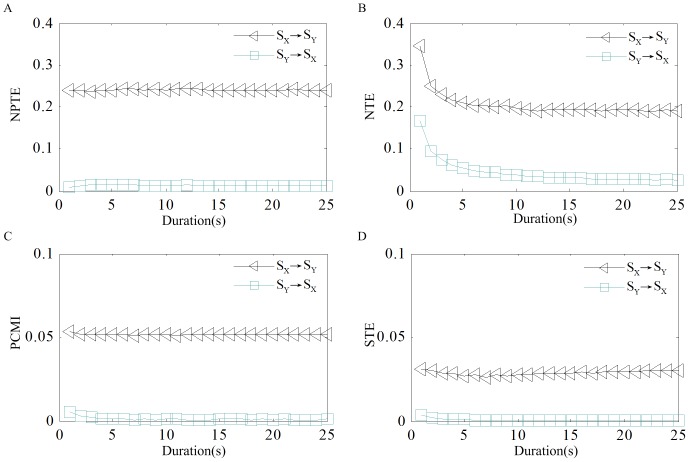
Estimated values for varying spike trains duration. (A) NPTE. (B) NTE. (C) PCMI. (D) STE.

We examine pairs of spike trains with different lengths to test whether the estimated values would change as a function of the data length. Given 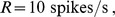 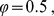 and 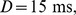 two spike trains are generated with a driving direction from 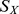 to 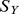. The duration ranges from 1s to 25s with a step of 1s. Figures 5(A–D) show the effects of increased data length on the estimation of NPTE, NTE, PCMI and STE respectively. It shows that the NPTE, PCMI and STE yields fairly consistent and robust estimates for various data lengths, meaning that they are largely independent of record length. This is rather valuable for practical applications where long recordings are not available. In comparison, the NTE shows significant reduction for shorter data length.

#### Time complexity

In real world scenarios, the speed of a method may be of considerable importance. Thus, it is necessary to compare the computational demand of these four methods. First, the STE and PCMI are both based on the technique of permutation and sliding time steps. However, the calculation of conditional mutual information is a little more complex than that of transfer entropy, which implies that the PCMI takes longer running time than the STE. Next, due to the procedure of bias removal and normalization, the NTE and NPTE need much more amount of calculation than the STE and PCMI. Moreover, the permutation analysis employed in the NPTE makes it more complicated than the NTE, and consequently its running takes more time.

#### Error of time delay estimation

**Figure 6:**
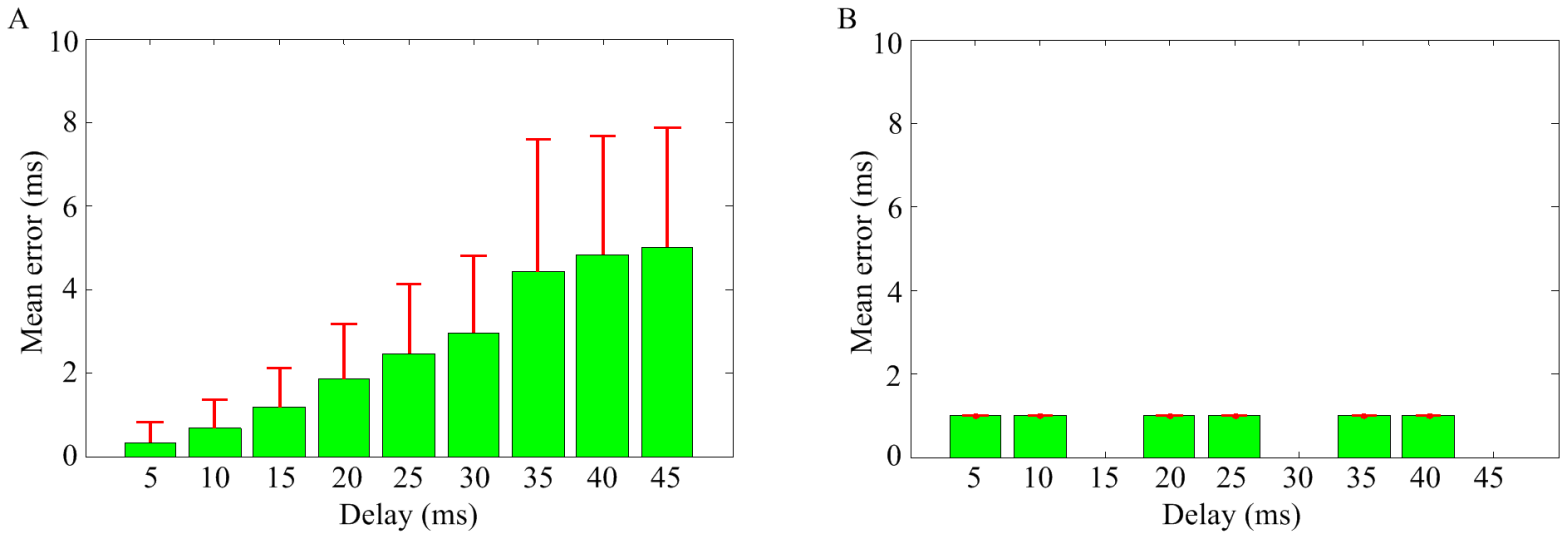
The error of estimation methods for different time delays. (A) NTE. (B) NPTE.

The methods mentioned in this paper are all able to estimate time delay of the causal interaction between spike trains. A typical distribution of axonal propagation delays between different pairs of cortical neurons is broad, ranging from several tenths to tens of milliseconds. In this study, we only investigate the performance for time delay ranging from 5 ms to 45 ms with a step of 5 ms. Given 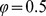 and 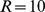
spikes/s, spike trains 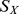 and 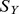
are generated. 500 realizations for each time delay are implemented by each method. We use Euclidean distance between simulation result and the time delay that used for constructing the model to quantify the error of each realization. The results are considered as outliers if they are larger than 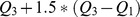 or smaller than 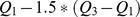, where 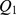 and 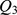 are the 25th and 75th percentiles, respectively. As shown in Figure 6A), the error and standard deviation of NTE estimation increases with the increase of time delay. In Figure 6(B), it can be seen that the error remains unchanged at 1 ms for the nine delays except for 15 ms, 30 ms and 45 ms. This is caused by the resolution of the NPTE (3 ms in this paper), which is related to the order. In other words, the NPTE is able to estimate precisely for time delays that are multiples of 3 ms, while with a fixed error of 1 ms for others. On the other hand, the PCMI and STE methods can always estimate the time delay without error and it is not plotted because of the zero values. Briefly, the NPTE is superior to the NTE for estimating time delay in terms of robustness and inferior to the PCMI and STE in resolution. The reasons are below. These four methods all use bins to discretize spike trains. In the NPTE and NTE, varying bins and normalization method are used to characterize the causal interaction. As mentioned above, large bins are likely to induce spurious increase of information transferred between spike trains, also bring about unstable and imprecise estimation of time delay. In this study, the NPTE method uses three bins simultaneously to overcome this defect with a little loss of resolution. On the other hand, the PCMI and STE, which employ a fixed bin (1 ms in this paper) and the sliding technique, can supply robust and precise estimation of time delay with a high resolution.

#### Effect of firing rate

**Figure 7:**
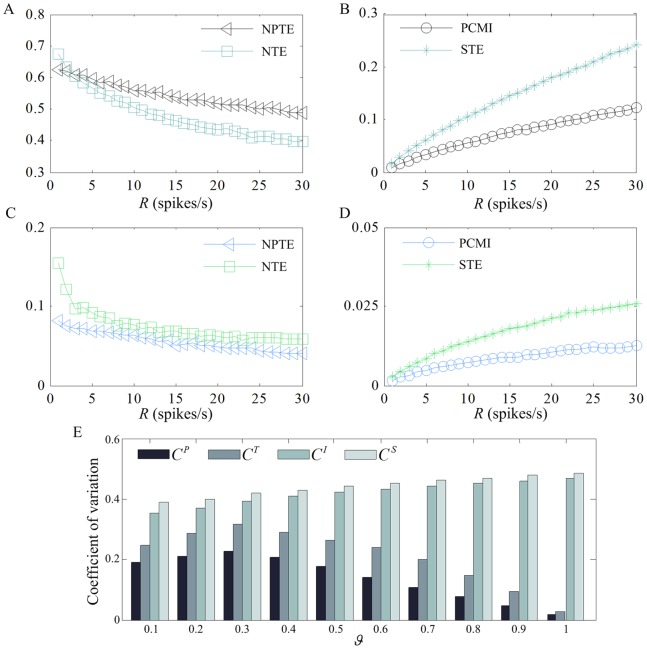
Effect of firing rate on the values estimated by different methods. (A) NPTE and NTE under 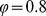. (B) PCMI and STE under 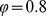. (C) NPTE and NTE under 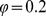. (D) PCMI and STE under 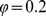. (E) Coefficients of variation for the four methods under different coupling strength.

Finally, we analyze the effect of firing rate on the performance of different methods. As we know, neurons may fire at different frequencies depending on their type and location. In this study, we make the firing rate *R* ranges from 1 spikes/s to 30 spikes/s with a step of 1 spikes/s. Given 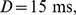
spike trains 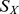 and 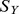 are generated. To investigate the effect at different coupling strength, we set 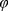 from 0.1 to 1 with a step of 0.1. In Figures 7(A–D), the estimated values of NPTE, NTE, PCMI and STE for different firing rates under 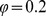 and 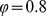 are plotted for illustration purpose. In either case, the PCMI and STE significantly increase with the increasing of firing rate. On the contrary, the NPTE and NTE decrease gradually as the firing rare increases. To explain explicitly, the effect is quantified by the coefficient of variation of the information flow at different firing rates from 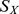 to 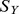
that estimated with different methods, including NPTE(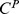), NTE(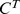), STE(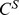) and PCMI(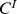). As shown in Figure 7(E), the outputs of the PCMI and STE methods are very similar, and the influence of firing rate on them is much more significant in comparison with the NPTE and NTE methods. Moreover, the NPTE is always less sensitive than the NTE to the firing rate. The underlying difference is that the NPTE is calculated in terms of ordinal patterns of spike trains, while the NTE uses the number of spikes in time intervals. The increase of the firing rate alters the joint distribution of ordinal patterns slightly, but modifies the distribution of spike numbers more significantly. As far as the PCMI and STE are concerned, the amount of information transferred between spike trains is estimated by means of the location of individual spikes in each spike train. Not surprisingly, it is proportional to the firing rate.

### Application to a Simulated Network

**Figure 8:**
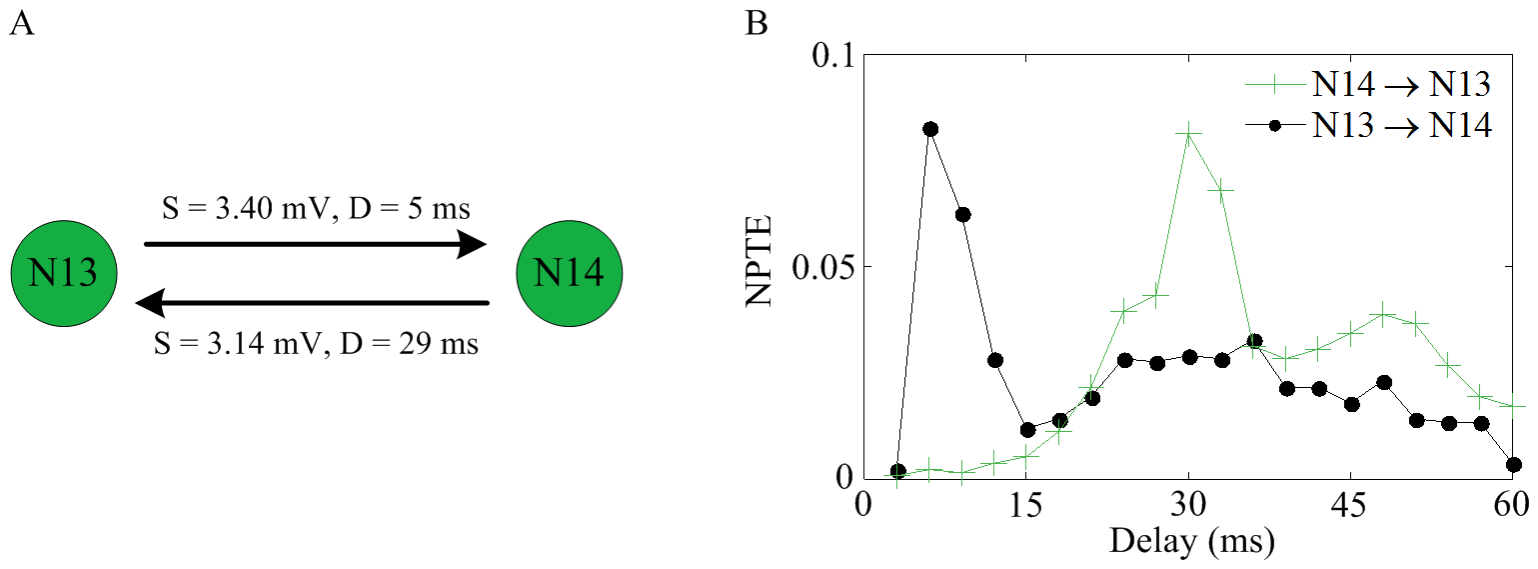
NPTE estimate in mutual coupling. (A) Two mutual coupled neurons sampled from a simulated network. (B) The NPTE estimate of connections on two directions.

**Figure 9:**
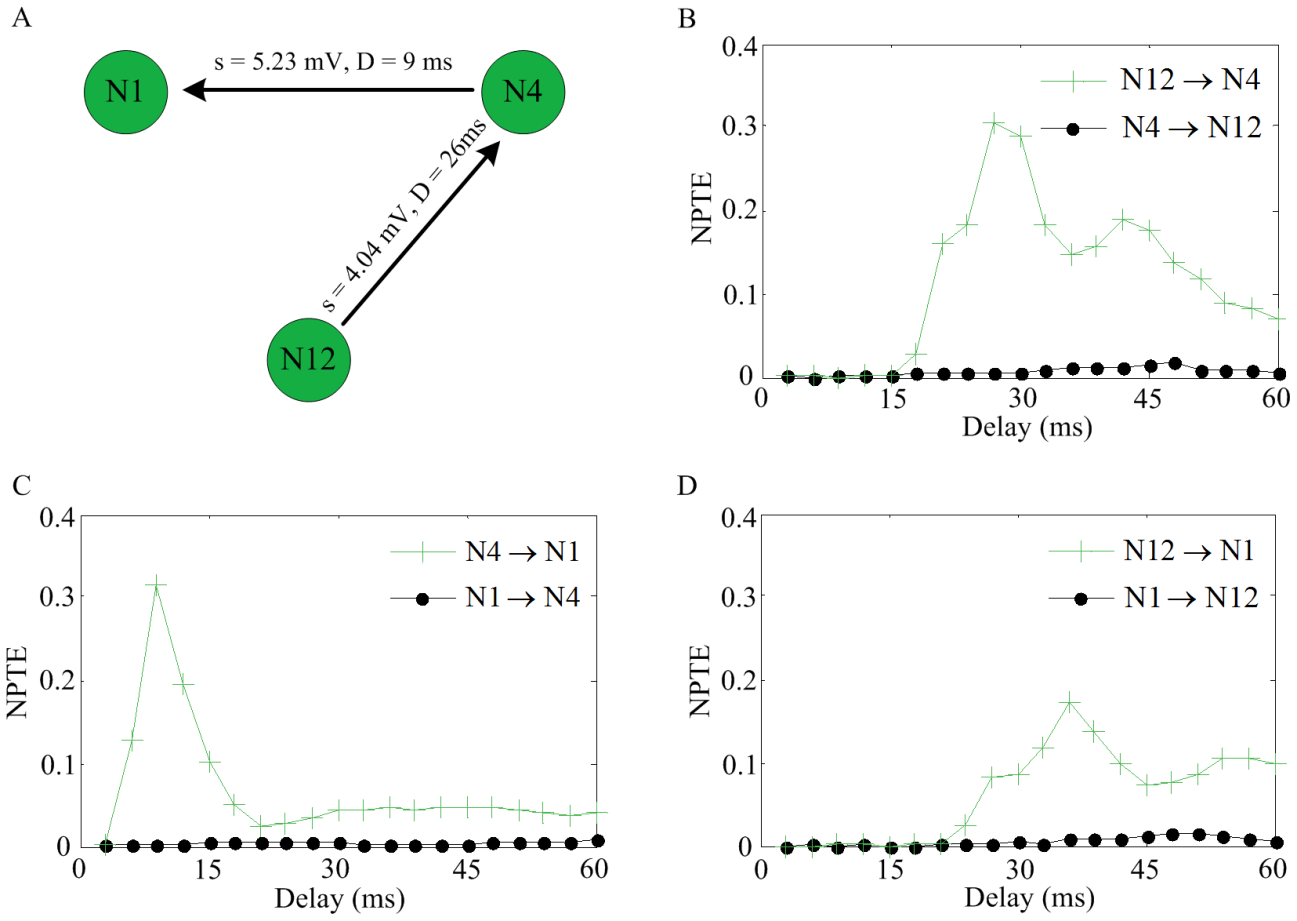
NPTE estimate in a network with three neurons. (A) Three neurons sampled from a simulated network. (B), (C) and (D) The pairwise NPTE estimate of connections on two directions.

We select two parts of a simulated network to investigate the performance of NPTE for characterizing mutual coupling and interactions between three neurons. As shown in Figure 8(A), two neurons N13 and N14 are connected through a mutual coupling with different strengths (denoted by s) and time delays (denoted by D) on two directions. In Figure 8, it can be seen that the maximum NPTE estimation from N13 to N14 occurs at the delay of 5 ms, while the maximum NPTE estimation appears at the delay of 29 ms in the opposite direction. This means that the NPTE method is able to describe the mutual coupling, with correct reflection of the strength but small error for the time delay estimate (1 ms for both directions). Next, we discuss how the NPTE measure performs in a sub-network of three neurons in which N12 is coupled via a synapse to N4, who is then connected to N1, as illustrated in Figure 9 (A). The NPTE estimate of these three pairwise coupling is plotted in Figure 9 (B–D). The causal relationships from N12 to N4 and from N4 to N1 are described effectively. At the same time, it can be also seen that there is a large value for the NPTE estimate from N12 to N1. In other words, the NPTE method shows an interaction raised by a third neuron. However, it should be noted that the time delay from N12 to N1 indicated by the NPTE method is 36 ms, which approximates to the summation of the delays from N12 to N4 and from N4 to N1 (19+5 = 34 ms). In addition, the maximum NPTE value from N12 to N1 is less than the values from N12 to N4 or from N4 to N1. Considering these two aspects, the causality appeared in the direction from N12 to N1 can be reckoned as a spurious one. Thus, it is reasonable to conclude that the NPTE method is able to distinguish real causalities from spurious ones. The underlying idea is explained as follows. For example, if there is a causal interaction from neuron X to neuron Y, then a portion of spikes of neuron Y will occur after some spikes of neuron X with a certain time delay. Suppose that this is hold for neuron Y and neuron Z. That is to say, some spikes of neuron Z will fire after certain spikes of neuron X, resulting in a large NPTE estimation that implies a spurious connection from neuron X to neuron Z. In fact, only the spikes of neuron Y that contribute to the causal relationship from neuron Y to neuron Z that also participate in the generation of causal connection from neuron X to neuron Y, can lead to a NPTE estimate about this spurious causal relationship. Consequently, this spurious NPTE estimate is less than the real ones, and the spurious delay is related to the two real delays. Thus, if this is observed in a network of three neurons, a spurious causality can be identified. To summarize, although the NPTE method cannot eliminate spurious interactions directly in the computation, it can tell whether interactions are direct or indirect by comparing the results of three neurons.

## Conclusion

We proposed a new measure, called as NPTE, to characterize the causal interactions between two neural spike trains. This method allows us to quantify the information flow between spike trains and estimate the time delay of the interaction with consistent and robust results for various data lengths. Comparing with the recently developed information theoretic methods, including NTE, STE and PCMI, the NPTE shows no more advantage on variation of coupling strength and independence of data length. However, the prominent advantage is that the NPTE is less sensitive to the firing rate of neurons than the others. In other words, the NPTE is a more reliable method to estimate the amount of information flow between neural spike trains with different firing rates. On the contrary, the STE and PCMI are significantly influenced by the firing rates. The immunity of the NTE against firing rate is close to the one of NPTE in some cases. However, the NPTE is superior to the NTE for estimating the time delay of the interaction between neurons. Although the resolution of NPTE is not as high as that of STE and PCMI, it is able to provide an accurate characterization about the time delay. Moreover, results of the two sub-networks embedded in a simulated network of 20 neurons show that the NPTE method can describe the mutual interactions or distinguish spurious causalities effectively. In addition, it should be noted that the NPTE takes more running time than that of the other three methods to gain the advantages. However, the speed of the algorithm can be improved by using faster CPU and larger memory or taking advantage of GPU, where the latter is a focus of our future works. In view of these aspects, the NPTE is a promising method for the analysis of causal interactions between spike trains.

## Supporting Information

File S1 **This contains three information theoretic methods for comparison.**
(DOC)
