# Supplementary material for: Estimating Temporal Causal Interaction between Spike Trains with Permutation and Transfer Entropy
Source: PLoS One. 2013 Aug 5;8(8):e70894. doi: 10.1371/journal.pone.0070894 (PMC3733844; doi:10.1371/journal.pone.0070894)
Supplement: File S1 — This contains three information theoretic methods for comparison. (DOC) [file pone.0070894.s001.doc]

**SUPPORTING MATERIAL**

**Estimating temporal causal interaction between spike trains with permutation and transfer entropy**

Zhaohui Li, Xiaoli Li

**Supporting details of the three information theoretic methods for comparison:**

1. Normalized transfer entropy (NTE)

Let $[t t+\tau_{f}]$ and $[t-\tau_{p} t]$ denote the upcoming time interval and past time interval, respectively, and $X^{F}$, $X^{P}$ and $Y^{F}$, $Y^{P}$ are the number of spikes of $S_{X}$ and $S_{Y}$ falling in the two intervals, then the transfer entropy from $S_{X}$ to $S_{Y}$ is defined as :

$$\xi_{X\to Y}=I\left(Y^{F};X^{P}|Y^{P}\right)=H\left(Y^{F}|Y^{P}\right)-H\left(Y^{F}|X^{P},Y^{P}\right)$$

To reduce the bias caused by the surrogate data that generated by randomly shuffling the inter-spike intervals, the normalized transfer entropy (NTE) is defined as:

$$\psi_{X\to Y}=\frac{\xi_{X\to Y}-\xi_{X\to Y}^{\text{shuffled}}}{H\left(Y^{F}|Y^{P}\right)}$$

The NTE is possibly less than zero when there is no causality between two spike trains. To restrict the NTE in the interval [0 1], we set up: $\psi=0$ if $\psi<0$.

1. Permutation conditional mutual information (PCMI)

To calculate the PCMI, two spike trains $S_{X}$ and $S_{Y}$are first converted to discretized sequences by using a fixed bin. Then, let $X=\left\{\hat{x}_{i}\right\}$ and $Y=\left\{\hat{y}_{i}\right\}$ denote the ordinal patterns of these two sequences respectively. Here, we choose the same order as the one used to compute the NPTE, i.e., 3. The processes to obtain *X* and *Y* is similar to that in the calculation of NPTE. The marginal probability distribution functions of *X* and *Y* are denoted as $p\left(x\right)$ and $p\left(y\right)$ and the joint probability function of *X* and *Y* is denoted as $p\left(x,y\right)$. Then, the entropy of *X* and *Y* can be defined as

$$H\left(X\right)=-\sum_{x\in X} p\left(x\right)\log p\left(x\right)$$

and

$$H\left(Y\right)=-\sum_{y\in Y} p\left(y\right)\log p\left(y\right)$$

The joint entropy $H\left(X,Y\right)$ of X and Y is

$$H\left(X,Y\right)=-\sum_{x\in X} \sum_{y\in Y} p\left(x,y\right)\log p\left(x,y\right)$$

The conditional entropy $H\left(X|Y\right)$ of X given Y is given by

$$H\left(X|Y\right)=-\sum_{x\in X} \sum_{y\in Y} p\left(x,y\right)\log p\left(x|y\right)$$

Then, the common information contained in both X and Y can be evaluated by the mutual information:

$$I\left(X;Y\right)=H\left(X\right)+H\left(Y\right)-H\left(X,Y\right)$$

To infer the causal relationship, the PCMI between X and Y can be calculated by the following equations

$$I_{X\to Y}^{\delta}=I\left(X;Y_{\delta}|Y\right)=H\left(X|Y\right)+H\left(Y_{\delta}|Y\right)-H\left(X,Y_{\delta}|Y\right)$$

and

$$I_{Y\to X}^{\delta}=I\left(Y;X_{\delta}|X\right)=H\left(Y|X\right)+H\left(X_{\delta}|X\right)-H\left(Y,X_{\delta}|X\right)$$

where $X_{\delta}$($Y_{\delta}$) is the future $\delta$ steps ahead of the process *X*(*Y*) [2,3].

1. Symbolic transfer entropy (STE)

In the computation of STE, a symbol is defined as the ordinal pattern of *m* data points, where *m* is the order used to construct motifs. To keep consistency, we set $m=3$in this paper. Given two symbol sequences $\left\{\hat{x}_{i}\right\}$ and $\left\{\hat{y}_{i}\right\}$ (we use the same notation as the PCMI, meaning that they are obtained in the same way), the STE can be defined as

$$S_{X\to Y}=\sum p\left(\hat{y}_{i+\delta},\hat{y}_{i},\hat{x}_{i}\right) \text{log}_{2}\frac{p\left(\hat{y}_{i+\delta}|\hat{y}_{i},\hat{x}_{i}\right)}{p\left(\hat{y}_{i+\delta}|\hat{y}_{i}\right)}$$

where the sum runs over all symbols and $\delta$ denotes a time step .

**References**

1. Gourévitch B, Eggermont J (2007) Evaluating Information Transfer Between Auditory Cortical Neurons. J Neurophysiol 97: 2533-2543.

2. Li Z, Ouyang G, Li D, Li X (2011) Characterization of the causality between spike trains with permutation conditional mutual information. Phys Rev E 84: 021929.

3. Palu M, Komárek V, Hrn í Z, těrbová K (2001) Synchronization as adjustment of information rates: Detection from bivariate time series. Phys Rev E 63: 46211.

4. Staniek M, Lehnertz K (2008) Symbolic transfer entropy. Phys Rev Lett 100: 158101.
